# Systematic Search for Evidence of Interdomain Horizontal Gene Transfer from Prokaryotes to Oomycete Lineages

**DOI:** 10.1128/mSphere.00195-16

**Published:** 2016-09-14

**Authors:** Charley G. P. McCarthy, David A. Fitzpatrick

**Affiliations:** Genome Evolution Laboratory, Department of Biology, Maynooth University, Maynooth, County Kildare, Ireland; Carnegie Mellon University

**Keywords:** *Phytophthora*, *Phytopythium*, *Pythium*, interdomain HGT, oomycota

## Abstract

While most commonly associated with prokaryotes, horizontal gene transfer (HGT) can also have a significant influence on the evolution of microscopic eukaryotes. Systematic analysis of HGT in the genomes of the oomycetes, filamentous eukaryotic microorganisms in the *Stramenopiles*-*Alveolates*-*Rhizaria* (SAR) supergroup, has to date focused mainly on intradomain transfer events between oomycetes and fungi. Using systematic whole-genome analysis followed by phylogenetic reconstruction, we have investigated the extent of interdomain HGT between bacteria and plant-pathogenic oomycetes. We report five putative instances of HGT from bacteria into the oomycetes. Two transfers were found in *Phytophthora* species, including one unique to the cucurbit pathogen *Phytophthora capsici*. Two were found in *Pythium* species only, and the final transfer event was present in *Phytopythium* and *Pythium* species, the first reported bacterium-inherited genes in these genera. Our putative transfers included one protein that appears to be a member of the *Pythium* secretome, metabolic proteins, and enzymes that could potentially break down xenobiotics within the cell. Our findings complement both previous reports of bacterial genes in oomycete and SAR genomes and the growing body of evidence suggesting that interdomain transfer from prokaryotes into eukaryotes occurs more frequently than previously thought.

**IMPORTANCE** Horizontal gene transfer (HGT) is the nonvertical inheritance of genetic material by transfer between different species. HGT is an important evolutionary mechanism for prokaryotes and in some cases is responsible for the spread of antibiotic resistance from resistant to benign species. Genome analysis has shown that examples of HGT are not as frequent in eukaryotes, but when they do occur they may have important evolutionary consequences. For example, the acquisition of fungal genes by an ancestral *Phytophthora* (plant destroyer) species is responsible for the large repertoire of enzymes in the plant-degrading arsenal of modern-day *Phytophthora* species. In this analysis, we set out to systematically search oomycete genomes for evidence of interdomain HGT (transfer of bacterial genes into oomycete species). Our results show that interdomain HGT is rare in oomycetes but has occurred. We located five well-supported examples, including one that could potentially break down xenobiotics within the cell.

## Abstract (precis)

Horizontal gene transfer (HGT) is the nonvertical inheritance of genetic material by transfer between different species. HGT is an important evolutionary mechanism for prokaryotes and in some cases is responsible for the spread of antibiotic resistance from resistant to benign species. Genome analysis has shown that examples of HGT are not as frequent in eukaryotes, but when they do occur they may have important evolutionary consequences. For example, the acquisition of fungal genes by an ancestral *Phytophthora* (plant destroyer) species is responsible for the large repertoire of enzymes in the plant-degrading arsenal of modern-day *Phytophthora* species. In this analysis, we set out to systematically search oomycete genomes for evidence of interdomain HGT (transfer of bacterial genes into oomycete species). Our results show that interdomain HGT is rare in oomycetes but has occurred. We located five well-supported examples, including one that could potentially break down xenobiotics within the cell.

## INTRODUCTION

Horizontal gene transfer (HGT), “the nongenealogical transfer of genetic material from one organism to another” (1), is most closely associated with antimicrobial resistance in bacteria. The cumulative effect of transfer events has had a significant impact on overall prokaryotic genome evolution. For example, it is estimated that up to 80% of genes in some prokaryote genomes underwent intradomain HGT at some point in their history (2). Interdomain transfer of genetic material between prokaryotes and eukaryotes has previously been understood in the context of endosymbiotic gene transfer, which has made a significant contribution to the evolution of eukaryotic genomes (3), most notably in the evolution of the mitochondrion in eukaryotes through an ancestral primary endosymbiosis event with a *Rickettsia*-like alphaproteobacterium and the evolution of the plastid in the *Archaeplastida* through ancestral primary endosymbosis with a cyanobacterium (4). However, there is a growing body of literature supporting the notion of the existence of HGT between prokaryotes and eukaryotes, and many nonendosymbiotic horizontal interdomain gene transfer events between bacteria and eukaryotes have been described (5). Numerous metabolic genes have been transferred into the genomes of parasitic microbial eukaryotes (6, 7). Over 700 bacterial genes are present across fungi, with a particular concentration in *Pezizomycotina* (8); 71 putative bacterial genes have been identified in *Hydra vulgaris* (9); and the plant-parasitic nematode *Meloidogyne incognita* secretes cell wall-degrading enzymes inherited from soil-dwelling *Actinomycetales* and the betaproteobacterium *Ralstonia solanacearum* (10).

The oomycetes are a class of microscopic eukaryotes placed in the diverse stramenopile (or heterokont) lineage within the *Stramenopiles*-*Alveolata*-*Rhizaria* (SAR) eukaryotic supergroup (11). Historically classified as fungi due to their filamentous growth and similar ecological roles, oomycetes can be distinguished from “true” fungi by a number of structural, metabolic, and reproductive differences (12). The present placement of the oomycetes within the stramenopile lineage, and, by extension, within the SAR supergroup, is supported by phylogenomic analyses of 18S rRNA and conserved protein and expressed sequence tag (EST) data, which also support the supergroup’s monophyly over previous configurations such as “chromalveolates” (13–16).

The most ecologically destructive orders within the oomycetes are the *Saprolegniales* order, whose member species are known as “cotton molds,” which includes marine and freshwater pathogens of fish, and the closely related and predominantly terrestrial plant-pathogenic orders *Peronosporales* and *Pythiales* (17). The *Pythiales* order includes members of the marine and terrestrial genus *Pythium*, necrotrophic generalistic causative agents of root rot and damping-off in many terrestrial plants (Table 1). Some species (*Pythium aphanidermatum* and *Pythium ultimum*) are found under high-temperature or greenhouse conditions, while others (*Pythium irregulare* and *Pythium iwayami*) are most virulent at lower temperatures (18). *Pythium ultimum* and *Pythium irregulare* have broad ecological host ranges, while *Pythium iwayami* and *Pythium arrhenomanes* display some preference for monocots (18, 19).

**TABLE 1:** Summary of host ranges of plant-parasitic oomycete species analyzed in this study^a^

Species	Host(s)
*Phytophthora capsici*	Curcubits (e.g., *Cucurbita pepo*)
*Phytophthora infestans*	*Solanaceae* (e.g., *Solanum tuberosum*)
*Phytophthora kernoviae*	*Fagus sylvatica*, *Rhododendron*
*Phytophthora lateralis*	*Chamaecyparis lawsoniana*
*Phytophthora parasitica*	Broad range, including *Nicotiana tabacum*
*Phytophthora ramorum*	Broad range, including *Quercus*, *Rhododendron*
*Phytophthora sojae*	*Glycine max*
*Phytopythium vexans*	Tropical forest species
*Pythium aphanidermatum*	Broad range, virulent at higher temperatures
*Pythium arrhenomanes*	Monocots
*Pythium irregulare*	Broad range, virulent at lower temperatures
*Pythium iwayami*	Monocots, virulent at lower temperatures
*Pythium ultimum* var. *sporangiiferum*	Broad range
*Pythium ultimum* var. *ultimum*	Broad range, virulent at higher temperatures

a Refer to the introduction for references.

The *Peronosporales* order includes the paraphyletic hemibiotrophic genus *Phytophthora*, whose member species exhibit both broad and highly specialized host ranges (Table 1). Generalistic *Pythophthora* species include *Phytophthora ramorum* and *Phytophthora kernoviae* (causing sudden oak death and dieback in many other plant species, particularly *Rhododendron* spp.), *Phytophthora parasitica* (causing black shank disease in a diverse range of plants), and *Phytophthora capsici* (causing blight and root rot in *Cucurbitaceae*, *Solanaceae*, and *Fabaceae*). Species with more specialized host ranges include *Phytophthora sojae* and *Phytophthora lateralis* (causing root rot in soybean and Port Orford cedar, respectively), and *Phytophthora infestans* (causing late blight in some *Solanaceae* spp., most notoriously in potato). The tropical plant pathogen *Phytopythium vexans* was previously classified in *Pythium* clade K (19), but that clade has since been reclassified into *Phytopythium*, a morphological and phylogenetic genus intermediate between *Phytopthora* and *Pythium* (20).

To date, large-scale systematic analysis of the influence of HGT on oomycete genome evolution has focused on intradomain transfer between fungi and oomycetes (21, 22). The most extensive study revealed up to 34 putative transfers from fungi to oomycetes, many of which were associated with enzymes involved in carbohydrate metabolism (23). Three of these genes had previously been transferred from bacteria to fungi (24). Few events of HGT between bacteria and oomycetes have been described in the literature, and most incidents of interdomain HGT have been discovered within the context of fungus-focused studies. However, recent analyses have shown that actinobacterial cutinase has orthologs in a number of *Phytophthora* species (25), with subsequent copy expansion in *Phytophthora sojae*. Disintegrins and endonucleases secreted by *Saprolegnia parasitica* appear to be bacterial in origin (26), and studies of the secretomes of *Saprolegniales* species *Achlya hypogyna* and *Thraustotheca clavata* revealed one ancestral endoglucanase and three genes specific to the *Saprolegniales* order which had been transferred from bacteria (27). As with other unicellular eukaryotes, some genes in *Phytophtora* involved in amino acid metabolism have been obtained via horizontal transfer from bacteria (28). Other studies have identified ancestral bacterial events of HGT within other stramenopile genomes (29) or in other lineages within the SAR supergroup (30–32).

In light of these previous studies of the influence of HGT in the evolution of the oomycetes, we undertook a systematic investigation focusing on the extent of bacterial transfer into the oomycetes. We analyzed 13 species from the plant-pathogenic genera *Pythium* and *Phytophthora*, as well as the recently reclassified species *Phytopythium vexans*, for genes with sufficient evidence for nonvertical inheritance from bacteria. Here, we report five recent transfers from bacteria into individual oomycete lineages, including what we believe to be the first descriptions of interdomain HGT involving *Pythium*.

## RESULTS AND DISCUSSION

### Analysis of bacterial HGT into *Phytophthora* and *Pythium.*

To investigate the extent of bacterial HGT into the oomycetes, we generated gene phylogenies for every oomycete protein sequence whose bidirectional homology analysis supported a recent transfer from bacteria to an oomycete species. Such phylogenies were generated with techniques that have previously identified multiple intradomain events of HGT between fungi and oomycetes (23): using OrthoMCL (33) to generate clusters of orthologous proteins, searching representative proteins against a large database using BLASTp (34), and generating maximum-likelihood phylogenetic reconstructions using PhyML (35). To reduce the chances of false-positive identification of putative HGT genes due to poor taxon sampling (36, 37), oomycete protein sequences were queried against a local database using BLASTp, with broad taxon sampling in the database across prokaryotes and eukaryotes (see Data Set S1 in the supplemental material). A total of 106 oomycete proteins were found to have a top database hit with a bacterial protein. Filtering for redundancy (due to multiple homologs in a single species, for example), 64 unique candidate maximum-likelihood HGT phylogenies with 100 bootstrap replicates (Table 2) were generated using PhyML with the best-fit model for each phylogeny chosen by ProtTest (38). Through our process of examination, we retained 25 phylogenies which satisfied our criteria (resolvable topology and adequate taxon sampling) (Table 2). Of these 25 phylogenies, 20 were ultimately discarded due to poor phylogenetic and bootstrap support or signal. Our phylogenies infer three types of bacterium-oomycete HGT within our candidate HGT phylogenies:

(i) Recent bacterial transfer into the *Pythium* or *Phytopythium* (*Pythium*/*Phytopythium*) lineage (1 individual example).
(ii) Recent bacterial transfer into the *Phytophthora* lineage (2 individual examples).
(iii) Recent bacterial transfer into the *Pythium* lineage (2 individual examples).

10.1128/mSphere.00195-16.10 Data Set S1  Excel sheet containing a list of the organisms in our local protein sequence database and also data for Pfam analysis of putative HGT genes. Download Data Set S1, XLSX file, 0.1 MB. Copyright © 2016 McCarthy and Fitzpatrick. 2016 McCarthy and Fitzpatrick This content is distributed under the terms of the Creative Commons Attribution 4.0 International license.

**TABLE 2:** Identification of putative bacterial HGT sequences in *Phytophthora*, *Pythium*, and *Phytopythium*

Genus	No. of intergenic bacterial hits	No. of OrthoMCL clusters (no. of sequences)	No. of OrthoMCL unclustered sequences	No. of maximum likelihood phylogenies	Putative no. of HGT sequences
*Phytophthora*	31	22 (28)	3	25	3
*Phytopythium*/*Pythium*	75	16 (59)	23	39	2

Each phylogeny was evaluated for other characteristics that might have led to reinforcement or rejection of our hypothesis that HGT had occurred. Gene characteristics such as GC content, exon number, and the sequence length of each oomycete gene arising from transfer in our phylogenies were calculated (see Table S1 in the supplemental material), and the results were compared to the average results determined for their corresponding genomes. Gene characteristics of bacterial homologs in potential donor species were also calculated (see Table S2). Similarly, the codon usage patterns of each *Phytophthora* and *Pythium*/*Phytopythium* genome were analyzed, and the patterns of each of the candidate genes potentially arising from HGT in each species were compared to the general pattern to see whether they were outliers. The codon usage patterns of the seed genes used to generate each phylogeny were also compared with the codon usage patterns of potential bacterial donors (not shown). None of these analyses were conclusive with respect to proving or disproving that horizontal inheritance of these genes had occurred. However, this is not uncommon for codon usage analyses as the codon usage of transferred genes is known to ameliorate to match that of the recipient genome (39). Sequence similarity and identity at the amino acid level between each seed HGT protein and a sister homolog from a potential bacterial donor were also investigated (see Table S3).

10.1128/mSphere.00195-16.6 Table S1  Genetic characteristics of putative bacteria HGT genes in oomycete genomes and comparison with the mean genetic characteristics of the corresponding genome. Refer to the corresponding figure for the phylogenetic tree. The seed gene is indicated in italics. Download Table S1, DOCX file, 0.02 MB. Copyright © 2016 McCarthy and Fitzpatrick. 2016 McCarthy and Fitzpatrick This content is distributed under the terms of the Creative Commons Attribution 4.0 International license.

10.1128/mSphere.00195-16.7 Table S2  Characteristics of potential sister genes to candidate HGT genes from potential donor bacterial species. Refer to the corresponding figure for the phylogenetic tree. Download Table S2, DOCX file, 0.02 MB. Copyright © 2016 McCarthy and Fitzpatrick. 2016 McCarthy and Fitzpatrick This content is distributed under the terms of the Creative Commons Attribution 4.0 International license.

10.1128/mSphere.00195-16.8 Table S3  Local protein-protein alignments of candidate HGT genes with homologs in potential donor species. Download Table S3, DOCX file, 0.01 MB. Copyright © 2016 McCarthy and Fitzpatrick. 2016 McCarthy and Fitzpatrick This content is distributed under the terms of the Creative Commons Attribution 4.0 International license.

To help ensure that none of our putative HGT families were in fact the product of bacterial contamination, the homology of each seed gene to its adjacent genes was investigated. In each of our five putative HGT families, we found that there was no obvious evidence of bacterial contamination along a source contig that resulted in false positives for bacterium-oomycete events of HGT (see Table S4 in the supplemental material). As we were also conscious of the risk of poor taxon sampling giving us false positives, we also compared the taxon sampling in our local database with the NCBI protein data. We queried each seed protein sequence against the NCBI’s nonredundant protein sequence database using BLASTp with an E value cutoff of 10^−20^, aligned homologs, and generated neighbor-joining phylogenies for each seed gene (not shown). Where the BLASTp data retrieved from NCBI mirrored our own local searches and the corresponding neighbor-joining phylogeny showed that the seed gene clearly grouped within an oomycete clade or a bacterial clade, we were satisfied that our taxon sampling had sufficiently covered all available protein data. All 5 of our candidate HGT genes satisfy these criteria.

10.1128/mSphere.00195-16.9 Table S4  Homology analysis of putative HGT sequences and adjacent sequences in oomycete genomes. Purple, *Phytophthora*; green, *Bacteria*; blue, *Aphanomyces invadans*; yellow, *Strongylocentrotus purpuratus*. Download Table S4, DOCX file, 0.02 MB. Copyright © 2016 McCarthy and Fitzpatrick. 2016 McCarthy and Fitzpatrick This content is distributed under the terms of the Creative Commons Attribution 4.0 International license.

**FIG 1:**
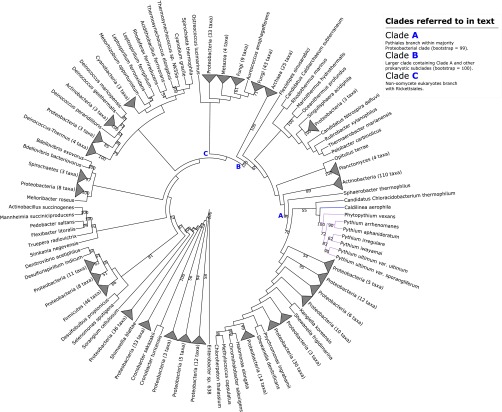
Maximum-likelihood phylogeny illustrating putative transfer of class II fumarase from *Caldilinea aerophila* into the *Phytopythium*/*Pythium* lineage. Clades A, B, and C referred to in the main text are highlighted. Selected bootstrap support values are shown at nodes. The corresponding full phylogenetic trees with detailed clades can be viewed in Fig. S1 in the supplemental material.

**FIG 2:**
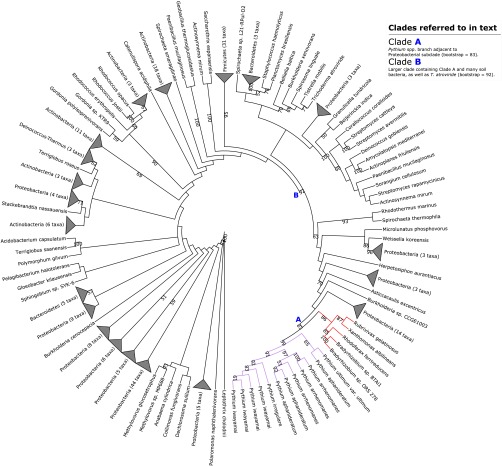
Maximum-likelihood phylogeny illustrating putative transfer of NmrA-like quinone oxidoreductase from *Proteobacteria* into *Pythium* spp. Clades A and B referred to in the main text are highlighted. Selected bootstrap support values are shown at nodes. The corresponding full phylogenetic trees with detailed clades can be viewed in Fig. S2 in the supplemental material. *T. atroviride*, *Trichoderma atroviride*.

**FIG 3:**
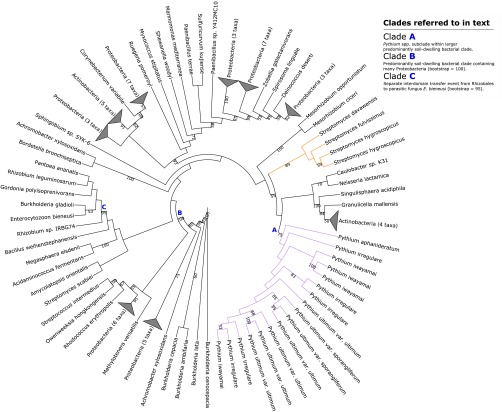
Maximum-likelihood phylogeny illustrating putative transfer of SnoaL-like polyketide cyclase from *Actinobacteria* into *Pythium* spp. Clades A, B, and C referred to in the main text are highlighted. Selected bootstrap support values are shown at nodes. The corresponding full phylogenetic trees with detailed clades can be viewed in Fig. S3 in the supplemental material.

**FIG 4:**
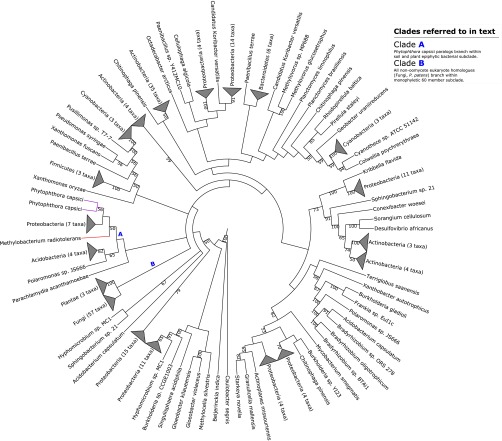
Maximum-likelihood phylogeny illustrating putative transfer of epoxide hydrolase from *Methylobacterium radiotolerans* into *Phytophthora capsici*. Clades A and B referred to in the main text are highlighted. Selected bootstrap support values are shown at nodes. The corresponding full phylogenetic trees with detailed clades can be viewed in Fig. S4A in the supplemental material.

**FIG 5:**
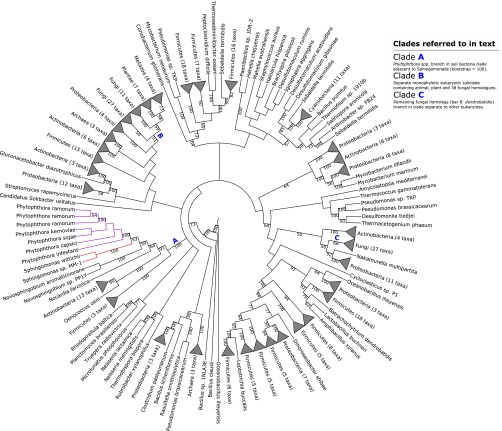
Maximum-likelihood phylogeny illustrating putative transfer of alcohol dehydrogenase from *Sphingomondales* into *Phytophthora* spp. Clades A, B, and C referred to in the main text are highlighted. Selected bootstrap support values are shown at nodes. The corresponding full phylogenetic trees with detailed clades can be viewed in Fig. S4B in the supplemental material.

We have identified five well-supported phylogenies that show putative events of HGT from bacterial species into the oomycetes. Three display topologies supporting a recent transfer into the *Pythium* or *Phytopythium* lineage (Fig. 1, 2, and 3), while the remaining two support a recent HGT into the *Phythophthora* lineage (Fig. 4 and 5). Below, we present and discuss each recent transfer individually, describing both the hypothesis for horizontal inheritance in each phylogenetic reconstruction and the functional characterization of each transferred gene family. We also compare the placement of the oomycete homologs in each of the five phylogenies with those of other eukaryotic homologs. This comparison is important as we expect transferred genes to violate the species phylogeny and transferred genes should form sister clades with bacterial species rather than their eukaryotic homologs. Each transfer is also summarized in Table 3.

10.1128/mSphere.00195-16.1 Figure S1  Maximum-likelihood phylogeny illustrating putative transfer of class II fumarase from *Caldilinea aerophila* into *Phytopythium*/*Pythium* lineage. Clades A, B, and C referred to in the main text are highlighted with purple, pink, and green branches, respectively. Bootstrap support values are shown at nodes. Download Figure S1, PDF file, 0.2 MB. Copyright © 2016 McCarthy and Fitzpatrick. 2016 McCarthy and Fitzpatrick This content is distributed under the terms of the Creative Commons Attribution 4.0 International license.

**TABLE 3:** Summary of each putative bacterium-oomycete HGT event

Tree	Seed species	Potential donor(s)	Identity (%)	Putative function	Secreted
Fig. 1	*Pythium ultimum*	*Caldilinea aerophila*	56.5	Class II fumarase	No
Fig. 2	*Pythium aphanidermatum*	*Proteobacteria*	54.0	NmrA-like quinone oxidoreductase	No
Fig. 3	*Pythium aphanidermatum*	*Actinobacteria*	58.6	SnoaL-like polyketide cyclase	Yes
Fig. 4	*Phytophthora capsici*	*Methylobacterium radiotolerans*	68.2	Epoxide hydrolase	No
Fig. 5	*Phytophthora capsici*	*Sphingomonas*	59.1	Alcohol dehydrogenase	No

### A putative class II fumarase distinct from *Rickettsia* class II fumarase in *Phytopythium vexans* and *Pythium* spp. originates from bacteria.

A protein in *Pythium ultimum* var. *sporangiiferum* (Table 3) was identified in our BLASTp homology searches as a candidate for an interdomain HGT event into oomycete species. The maximum-likelihood phylogeny of this protein family was generated from a family containing 550 homologs, with an LG+I+G+F substitution model (Fig. 1). A total of 16 bacterial phyla were present in this reconstruction, among which *Proteobacteria* and *Actinobacteria* were by far the most extensively represented. A total of 26 archaeal homologs were also present, of which all except a “*Candidatus* Caldiarchaeum subterraneum” sequence form a monophyletic clade. Across the eukaryotes, homologs are present in fungi, animals, green algae, and the stramenopiles.

Our phylogenetic reconstruction shows a monophyletic *Pythium*/*Phytopythium* clade within a large, predominantly proteobacterial clade with 99% bootstrap support, adjacent to a homolog from the filamentous *Chloroflexi* species *Caldilinea aerophila* (Fig. 1, clade A). Further back along the tree, this greater subclade branches deep within a large prokaryotic clade with 100% bootstrap support and contains three major subclades: the aformentioned majority-proteobacterial subclade containing *Pythium* and *Phytopythium* orthologs, a halophilic archaeal subclade, and a large actinobacterial subclade containing 110 homologs (Fig. 1, clade B). Elsewhere, all nonoomycete eukaryote homologs (with the exception of an adjacent sequence from the microscopic green alga *Ostreococcus lucimarinus*) are placed in a monophyletic eukaryote clade containing 52 fungal homologs, 4 animal homologs, and a homolog from the stramenopile alga *Aureococcus anophagefferns* adjacent to a clade containing 19 homologs from the alphaproteobacterial *Rickettsia* genus (Fig. 1, clade C). The neighbor-joining tree constructed from the BLAST homology search of the seed sequence against the NCBI’s database places the seed deep within a large prokaryotic clade containing *Proteobacteria*, *Actinobacteria*, and halophilic and methanogenic archaea, in a gammaproteobacterial subclade similar to what we observed in our phylogenetic reconstruction (not shown).

Sequence analysis of the seed gene and its flanking genes in the *Pythium ultimum* var. *sporangiiferum* genome did not return any obvious evidence of bacterial contamination; the top hit of the seed protein sequence against the NCBI database was a *C. aerophila* sequence, but the top hits of both flanking protein sequences were *Phytophthora parasitica* homologs (see Table S4 in the supplemental material). BLAST homology searches against the NCBI database found that the seed sequence shared sequence similarity with many bacterial class II fumarases, and Pfam analysis of the sequence identified two lyase domains and the characteristic *fumC* C terminus of a class II fumarase-like sequence (see Data Set S1). InterProScan analysis identified further fumarase protein sequence signatures (see Data Set S1). Fumarase, also known as fumarate hydratase (EC 4.2.1.2), is an enzyme that catalyzes the reversible hydration of fumarate to (S)-malate in the mitochondrion in eukaryotes, as a component of the tricarboxylic acid cycle (40), and promotion of histone H3 methylation and DNA repair in the cytosol (41). There are two classes of fumarase: the heat-labile dimeric class I fumarases encoded by *fumA* and *fumB* found in prokaryotes and the heat-stable tetrameric class II fumarase encoded by *fumC* found in both prokaryotes and eukaryotes (42). While associated with mitochondrial function in eukaryotes, class II fumarases with distinct evolutionary histories have been detected in amitochondriate trichomonads (43).

The nature of the conserved function of the gene encoding class II fumarases in eukaryotic respiration would suggest that this gene had arisen in the nuclear genome of *Pythium* and *Phytopythium* by endosymbiotic gene transfer from the mitochondrial genome (44) and hence was not a product of recent transfer. To investigate the relationship between this putative horizontally transferred fumarase and other potential fumarase orthologs in the oomycetes, we aligned the seed *Pythium ultimum* var. *sporangiiferum* sequence against 20 known oomycete and 230 other eukaryote and prokaryote class II fumarase sequences. Sequence and phylogenetic analysis showed that it branches as an outgroup in the corresponding phylogeny (not shown), suggesting that it is not an ortholog of the endosymbiotic oomycete class II fumarase. It seems most parsimonious to suggest, therefore, that this fumarase protein in *Pythium* and *Phytopythium vexans* is a class II fumarase distinct from endosymbiotic class II fumarase and arose by a completely separate transfer event, possibly with *C. aerophila* or another *Chloroflexi* species (*Sphaerobacter thermophilus*, for example) (Fig. 1). An interesting aspect of this phylogeny is the presence of a homolog from *Phytopythium vexans* branching with *Pythium* species and the absence of *Phytophthora* homologs in the phylogeny. *Phytopythium vexans*, along with other members of what was once *Pythium* clade K, was reclassified to the morphological intermediate genus *Phytopythium*, based on molecular evidence, with ribosomal large subunit (LSU), internal transcribed spacer (ITS), and mitochondrial cytochrome oxidase 1 (CO1). Furthermore, the resultant phylogenetic data grouped *Phytopythium* and *Phytophthora* as sister taxa with strong bootstrap support (20). This would suggest that the ancestor of the *Phytophthora*, *Phytopythium*, and *Pythium* species obtained a bacterial copy of the class II fumarase and that it was subsequently lost in the *Phytophthora* clade. Alternatively, if we assume that rare events of HGT can act as phylogenetic markers (3), it is plausible that *Phytopythium* and *Pythium* are in fact more closely related to one another, to the exclusion of *Phytophthora* species. This observation challenges the phylogeny derived from traditional phylogenetic markers (20), and we suggest that the relationships between these groups warrant further examination.

### A putative proteobacterial NmrA-like oxidoreductase is present in multiple *Pythium* species.

A *Pythium aphanidermatum* gene (Table 3) was identified in our homology searches as a candidate for bacterial HGT into an oomycete species. The maximum-likelihood phylogeny of this gene was constructed from a gene family containing 258 homologs, with an LG+I+G+F substitution model (Fig. 2). Among these homologs, 95% (245 of 258) were bacterial, representing 10 different phyla. The majority of bacterial homologs were from *Proteobacteria*, *Actinobacteria*, or *Firmicutes* species. Of the 13 eukaryote homologs present, 12 were from the oomycetes and 1 was from the fungal species *Trichoderma viride* (Fig. 2).

10.1128/mSphere.00195-16.2 Figure S2  Maximum-likelihood phylogeny illustrating putative transfer of NmrA-like quinone oxidoreductase from *Proteobacteria* into *Pythium* spp. Clades A and B referred to in the main text are highlighted with purple and pink branches, respectively. Bootstrap support values are shown at nodes. Download Figure S2, PDF file, 0.05 MB. Copyright © 2016 McCarthy and Fitzpatrick. 2016 McCarthy and Fitzpatrick This content is distributed under the terms of the Creative Commons Attribution 4.0 International license.

In our reconstruction, homologs (12 in total) from each *Pythium* species except *Pythium ultimum* var. *sporangiiferum* formed a monophyletic subclade (99% bootstrap support) within a 70-member clade with 92% bootstrap support. Every other member of this clade except *Trichoderma viride* was bacterial. Around 30 members of this clade, many of which were soil-dwelling *Rhizobales*, were proteobacterial (Fig. 2, clade B). The *Pythium* subclade branches with 83% bootstrap support beside a small proteobacterial subclade that includes two nitrogen-fixing species in *Bradyrhizobium* and *Xanthomonas albilineans*, the causative agent of leaf scald disease in sugarcane (45) (Fig. 2, clade A). Homology analysis of the seed sequence and its flanking sequences in the *P. aphanidermatum* genome found no obvious evidence of bacterial contamination, and the seed sequence was most closely related to a *Rubrivivax gelatinosus* sequence; however, flanking genes had top hits from *Phytophthora infestans* (see Table S4 in the supplemental material). The neighbor-joining phylogeny generated from BLAST homology searches of the seed sequence against the NCBI’s protein database also placed the seed sequence adjacent to a large proteobacterial clade (not shown).

BLAST homology searches against the NCBI database found that the seed sequence shared homology with bacterial nucleotide-sugar epimerases and NAD(P)-binding proteins. Pfam analysis of the sequence found the characteristic Rossmann fold of NAD(P)-binding proteins (see Data Set S1 in the supplemental material), while InterProScan analysis found NmrA-like family and quinone oxidoreductase 2 subfamily PANTHER signatures (see Data Set S1). NmrA is a NAD(P)-binding negative transcriptional regulator, involved in the regulation of nitrogen metabolite repression (NMR) genes in fungi, which suppress metabolic pathways for secondary nitrogen sources when preferred sources like ammonium and glutamine are available (46). Such a metabolic system has not been described in oomycetes to date. The PANTHER quinone oxidoreductae subfamily (47) to which this transferred gene belongs (PTHR14194:SF73) includes eukaryotic orthologs from *Pezizomycotina*, *Monosiga brevicollis* and *Dictyostelium* spp., *Phytophthora infestans* and *Physcomitrella patens*, and bacterial orthologs from multiple lineages. Among these orthologs is *qorB* in *Escherichia coli* K-12, which has redox activity on NAD(P)H using quinone as an acceptor (48).

Our phyogenetic reconstruction of this *Pythium aphanidermatum* gene supports the hypothesis of the transfer of this gene into *Pythium* spp. from a soil-dwelling proteobacterium (Fig. 2), either the phototrophic betaproteobacterial species *Rhodoferax ferrireducens*/*Rubrivivax gelatinosus* or the phytopathogenic gammaproteobacterium *Xanthomonas albilineans*. Species related to *X. albilineans* and *R. ferrireducens*, within *Xanthomonadales* and *Comamonadaceae*, respectively, have been identified in previous studies as endohyphal bacteria, hypha-dwelling endosymbionts of endophytic fungi (49, 50). It is not currently known whether such bacteria can also inhabit the hyphae of oomycetes and thus consequently provide favorable conditions for potential interdomain HGT. This transferred gene may be a NAD(P)H-binding quinone oxidoreductase (EC 1.6.5.2) and potentially has cytosolic redox activity in *Pythium* spp.

### SnoaL-like proteins from soil-dwelling bacteria are putative members of the secretome of multiple *Pythium* species.

A second gene from *P. aphanidermatum* (Table 3) was identified in our BLASTp homology searches as a candidate for bacterial HGT into an oomycete species. The maximum-likelihood phylogeny of this gene was generated from a gene family containing 103 homologs constructed with a WAG+I+G substitution model (Fig. 3). Seven bacterial phyla are present in this reconstruction, along with *Pythium* and the fungal parasite *Enterocytozoon bieneusi*, and 53% of the homologs (55 of 103) come from proteobacterial species.

10.1128/mSphere.00195-16.3 Figure S3  Maximum-likelihood phylogeny illustrating putative transfer of SnoaL-like polyketide cyclase from *Actinobacteria* into *Pythium* spp. Clades A, B, and C referred to in the main text are highlighted with purple, pink, and green branches, respectively. Bootstrap support values are shown at nodes. Download Figure S3, PDF file, 0.03 MB. Copyright © 2016 McCarthy and Fitzpatrick. 2016 McCarthy and Fitzpatrick This content is distributed under the terms of the Creative Commons Attribution 4.0 International license.

The maximum-likelihood phylogenetic reconstruction places 17 *Pythium* homologs (with multiple paralogs in each species except *P. aphanidermatum* and no homolog in *P. arrhenomanes*) deep within a 93-member clade containing many typical soil-dwelling proteobacterial and actinobacterial species (Fig. 3, clade B) with 100% bootstrap support. The *Pythium* subclade (Fig. 3, clade A) is adjacent to a clade containing four orthologs from *Mycobacterium smegmatis*. The only other eukaryote homolog in our analysis (*E. bieneusi*) is placed in a separate subclade containing *Rhizobales* species with 95% bootstrap support, indicative of a separate independent HGT event (Fig. 3, clade C). Homology analysis of the seed sequence and its adjacent sequences returned no evidence of bacterial contamination. The sequences of both flanking genes are homologous to sequences in other oomycetes, and the seed sequence’s highest degree of homology was with a *Streptomyces yerevanensis* sequence (see Table S4 in the supplemental material).

BLAST homology searches of the seed sequence found numerous instances of homology with bacterial SnoaL-like polyketide cyclases. Pfam and InterProScan analysis of the sequence identified two SnoaL-like domains and a number of signal peptide signatures within the N-terminal domain (see Data Set S1 in the supplemental material). Polyketide cyclases are enzymatic components of the synthesis of aromatic polyketide compounds from carboxylic acids in bacteria and fungi. Polyketides are best characterized by the medicinally useful secondary metabolites produced by various *Actinobacteria* genera, such as the antitumourigenic anthracyclines from *Streptomyces* species (51). Biochemically, polyketide cyclases catalyze the intramolecular cyclization of poly-β-ketone chain intermediates to form the core planar polyaromatic structures of polyketides, which are then subject to later functionalization. In the biosynthesis of the anthracycline nogalamycin in *Streptomyces nogalater*, the polyketide cyclase SnoaL (EC 5.5.1.26) catalyzes ring closure of a polyaromatic nogalamycin precursor through aldol condensation (52).

The maximum-likelihood phylogenetic reconstruction of this transfer event appears to support the transfer of this putative SnoaL-like protein into a *Pythium* ancestor from a proteobacterial or actinobacterial donor (Fig. 3)*.* Similarly, the neighbor-joining tree generated from the homology search against NCBI’s nonredundant database places the *P. aphanidermatum* seed sequence within a large proteobacterial and actinobacterial clade (not shown). The SignalP (53) and TargetP (54) analyses both indicated that the protein contains a 25-reside-long signal peptide sequence at its N terminus with a discrimination score (used to distinguish between signal and nonsignal peptides) well above the default cutoff value and thus identified the protein as part of the secretome of *P. aphanidermatum*. Therefore, this putative SnoaL-like protein may have arisen in *Pythium* species through horizontal transfer from an *Actinobacteria* species and may be a putative component of the secretome of *Pythium* species. It is worth noting that no polyketide synthase genes have been detected in model *Phytophthora* genomes and that, in general, oomycetes rely more on toxic effector proteins than on toxic small-molecule secondary metabolites for necrotrophic growth (55, 56). The presence of this putative SnoaL-like protein in multiple copies in most of the *Pythium* species that we investigated suggests an additional method of phytopathogenic infection which may be novel to *Pythium* or which may have been subsequently lost in *Phytophthora*.

### A putative hydrolase from xenobiotic-degrading rhizosphere proteobacteria is present in *Phytophthora capsici.*

A gene from *Phytophthora capsici* (Table 3) was identified in our BLASTp homology searches as a candidate for bacterial HGT. A maximum-likelihood phylogeny was generated from 253 homologs using a WAG+G substitution model. Eight bacterial phyla are represented in our reconstruction, with the majority of homologs coming from either proteobacterial or actinobacterial species. A total of 57 fungal homologs and 3 paralogs from *Physcomitrella patens* (earthmoss) form a monophyletic eukaryotic clade (Fig. 4, clade B). Our maximum-likelihood phylogenetic tree placed two homologs from *P. capsici* adjacent to a homolog from the alphaproteobacterium *Methylobacterium radiotolerans* within a bacterial clade containing *Acidobacteria* and a number of soil-borne or plant-epiphytic *Proteobacteria* (Fig. 4, clade A). BLASTp analysis aligned the seed sequence with an ortholog from the nitrogen-fixing proteobacterium *Azotobacter vinelandii*. As there is only one *Phytophthora* species represented in this phylogeny, we carefully examined the sequence of the contig to rule out a bacterial contamination artifact in the *P. capsici* genome. All flanking genes were from *Phytophthora* spp., thereby giving us confidence that this represents a bona fide HGT event (see Table S4 in the supplemental material). Furthermore, the phylogeny generated after homology searches against the NCBI database placed the seed sequence within a large proteobacterial clade (not shown).

10.1128/mSphere.00195-16.4 Figure S4  (A) Maximum-likelihood phylogeny illustrating putative transfer of epoxide hydrolase from *Methylobacterium radiotolerans* into *Phytophthora capsici*. Clades A and B referred to in the main text are highlighted with purple and green branches, respectively. Bootstrap support values are shown at nodes. (B) Maximum-likelihood phylogeny illustrating putative transfer of alcohol dehydrogenase from *Sphingomondales* into *Phytophthora* spp. Clades A, B, and C referred to in the main text are highlighted with purple, light green, and dark green branches, respectively. Bootstrap support values are shown at nodes. Download Figure S4, PDF file, 7.6 MB. Copyright © 2016 McCarthy and Fitzpatrick. 2016 McCarthy and Fitzpatrick This content is distributed under the terms of the Creative Commons Attribution 4.0 International license.

As the levels of bootstrap support for many of the more derived branches and clades in our phylogeny, including the bacterial clade containing *P. capsici* homologs, were weak (<50%), we generated a median phylogenetic network of all splits in the set of individual bootstrap trees generated by PhyML in our reconstruction using a consensus network method in SplitsTree (57). This consensus network (see Fig. S5 in the supplemental material) places the two *P. capsici* homologs at the base of the large monophyletic bacterial clade, clearly separate from the fungal and plant homologs. With this analysis, we were satisfied that the phylogeny represented a bona fide bacterium-oomycete HGT event.

10.1128/mSphere.00195-16.5 Figure S5  Median phylogenetic network of all splits in the set of individual bootstrap trees generated by PhyML in our hydrolase phylogeny (Fig. 4). The network was reconstructed using a consensus network method in SplitsTree (57). Download Figure S5, PDF file, 0.7 MB. Copyright © 2016 McCarthy and Fitzpatrick. 2016 McCarthy and Fitzpatrick This content is distributed under the terms of the Creative Commons Attribution 4.0 International license.

BLAST homology searches of the seed sequence against the NCBI database indicated that the sequence was homologous to those associated with bacterial hydrolases. Pfam analysis found a large α/β hydrolase fold domain present in the sequence, and InterProScan analysis returned a number of α/β hydrolase family PANTHER signatures, as well as epoxide hydrolase PRINTS (58) signatures, across the sequence (see Data Set S1 in the supplemental material). Epoxide hydrolases (EC 3.3.2.3) catalyze the dihydroxylation of epoxide residues to diols and are among the members of a number of protein families that contain an α/β hydrolase fold (59). Bacterial epoxide hydrolases are capable of degradation of xenobiotic organic compounds (60, 61). The structurally related haloalkane dehalogenases (EC 3.8.1.5), which can hydrolyze toxic haloalkanes into their corresponding alcohol and organic halide components in the cytosol, are widespread in soil bacteria (62). It is interesting that strains of *M. radiotolerans* isolated from *Cucurbita pepo* roots, which is also a target for *P. capsici*, are capable of degrading xenobiotic 1,1-*bis*-(4-chlorophenyl)-2,2-dichloroethene (DDE) (63). DDE is a highly toxic and highly recalcitrant major metabolite of the degradation of the toxic organochloride pesticide 1,1,1-trichloro-2,2-bis(p-chlorophenyl)ethane (DDT), which saw widespread use for most of the 20th century (64).

Our maximum-likelihood phylogenetic reconstruction suggests that this putative hydrolase gene, which has two copies in *P. capsici*, arose through horizontal transfer from soil-dwelling bacteria, potentially from *M. radiotolerans* (Fig. 4). Homology and functional analysis of the seed HGT gene indicates that these two paralogs contain hydrolase folds. The two paralogs in *P. capsici* are somewhat dissimilar at the nucleotide level; one appears to contain both peptidase and α/β hydrolase domains and is far more exonic than the seed HGT gene (see Table S1 in the supplemental material). This putative transferred gene may have a potential cytosolic role in the degradation of toxic xenobiotic compounds in *P. capsici*. To date, descriptions of xenobiotic degradation or resistance in oomycetes have been sparse in the literature; what is known is that few oomycete cytochrome P450 proteins (CYPs) appear to be involved in xenobiotic degradation compared with fungal CYPs (65, 66) and that *Phytophthora infestans* has far a lower proportion of major facilitator superfamily (MFS) transport proteins involved in efflux than many fungal type species do (67). As such, this acquisition may be a novel event in the context of plant-parasitic oomycete genome evolution.

### Sphingomonadale alcohol dehydrogenase is present in five *Phytophthora* species.

A second *P. capsici* gene (Table 3) was identified in our BLASTp homology searches as a candidate for interdomain HGT. Our phylogenetic reconstruction used 358 homologs with an LG+I+G substitution model (Fig. 5). Nine bacterial phyla are represented in this reconstruction, the majority of which are homologs from *Firmicutes* species, and 23% (84 of 358) of the homologs are of eukaryotic origin. Animal, plant, and 38 fungal homologs form a eukaryote monophyletic clade (Fig. 5, clade B). A total of 27 of the remaining 28 fungal homologs form a separate subclade (Fig. 5, clade C) almost entirely comprised of homologs from *Ascomycotes* except for two paralogs from the *Basidiomycota* species *Phlebiopsis gigantea*, while *Batrachochytrium dendrobatidis* is placed within an adjacent *Firmicutes* subclade.

Our maximum-likelihood phylogeny inferred a monophyletic *Phytophthora* subclade with seven homologs from five species (excluding *P. lateralis* and *P. parasitica*) within an alphaproteobacterial *Sphingomonadale* subclade with 100% bootstrap support (Fig. 5, clade A). Homology data for the seed sequence and its adjacent sequences within the *P. capsici* genome from JGI showed no obvious evidence of bacterial contamination at the genomic level, as neither of the flanking genes was bacterial in origin (see Table S4 in the supplemental material).

BLAST homology searches of the seed sequence returned hits from many bacterial alcohol dehydrogenase proteins. Pfam and InterProScan analysis of the seed sequence found that it contained the hallmark signatures of a medium-chain Zn^2+^-containing alcohol dehydrogenase: an N terminus containing the conserved Zn^2+^ active site, the conserved GroES-like fold, and the NAD(P)-binding Rossmann fold (see Data Set S1 in the supplemental material). Alcohol dehydrogenases (EC 1.1.1.1) catalyze the NAD(P)-dependent reversible oxidation of alcohols to aldehydes or ketones. In most prokaryotes, fungi, and plants, alcohol dehydrogenase is responsible for the reversed regeneration of NAD^+^ in fermentation for glycolysis from the reduction of NADH and acetaldehyde to NAD^+^ and ethanol. The high concentration of *Firmicutes* and fungal homologs in our reconstruction underlines the enzyme’s important role in anaerobic *Clostridia* and fungi. Previous EST analysis of *P. sojae* infection of soybean found abundant matches for alcohol dehydrogenase genes, among other intermediary metabolic genes differently expressed in host tissue, suggesting that alcohol fermentation is an important part of the catabolism of *P. sojae* in the early stages of growth inside host tissue (68).

The maximum-likelihood phylogenetic reconstruction performed for these putative *Phytophthora* alcohol dehydrogenase proteins supports the notion of a putative transfer from the alphaproteobacterial *Sphingomonadales* (Fig. 5). Similarly, the phylogeny generated in querying the seed sequence against the NCBI’s nonredundant protein database placed the seed sequence within a small *Phytophthora* subclade that was found within a larger *Sphingobium* and *Novosphingobium* clade (not shown). We therefore propose that this alcohol dehydrogenase, found in a number of *Phytophthora* species, arose in these species via recent transfer of the gene from *Sphingomonadales*.

### Impact and extent of bacterial genes in oomycete evolution.

Using stringent criteria, our analysis has found five putative gene families in oomycete species that have been acquired through horizontal transfer from bacteria. All five transfer events involve genes coding for proteins with putative enzymatic functions in their respective species; some of our findings, particularly with respect to the putative epoxide hydrolase gene in *Phytophthora capsici*, appear to represent novel evolutions, and some, particularly with respect to the fumarase and alcohol dehydrogenase families, complement those found in other analyses of HGT in oomycete genomes. Many of the inter- and intradomain HGT gene families identified in oomycete genomes to date are proteins with a putative carbohydrate metabolism function (16); in the most extensive study of HGT into oomycete genomes to date, Richards et al. (23) found 13 secreted proteins among the 34 potential fungal events of HGT in oomycetes that could be assigned with such a function. Of the seven bacterial events of HGT identified in oomycete species prior to our analysis (16), most were found in analyses of *Saprolegniales* species (21, 22) and, where function could be assigned, were thought to be involved in carbohydrate metabolism also.

The bacterially derived enzymes identified in oomycete species could have potentially found themselves more amenable to transfer and subsequent retention in oomycete genomes due to their relative low connectivity within a protein-protein interaction network, a significant factor in the influence of the “complexity hypothesis” on HGT (69, 70). The relatively low number of bacterium-oomycete events of HGT identified in this study and elsewhere in the literature, in comparison with other such studies of interdomain HGT, in fungi (8), for example, may be partially explained by the paucity of oomycete genomic data overall and the lack of data for more basal lineages in particular (12). Furthermore, our analysis was designed specifically to identify recent events of HGT in individual plant-parasitic oomycete lineages, as opposed to ancient transfers into the class as a whole or even into the greater stramenopiles group. Future analyses, facilitated by a greater amount of oomycete genomic data, may identify more instances of either bacterium-oomycete HGT to specific lineages or ancient transfers into the class.

### Conclusions.

Using methods similar to those that have previously identified intradomain HGT between fungi and *Phytophthora* (23), we have identified five interdomain events of HGT between bacteria and plant-pathogenic oomycetes (Table 3). Of the five putative bacterium-oomycete HGT genes that we have identified (Table 3), one has signal peptide signatures and subcellular localization matches that indicate that it is part of the oomycete secretome. The putative SnoaL-like protein may be a secreted transport protein or involved in production of other components of the *Pythium* secretome. A class II fumarase distinct from the endosymbiosis-derived fumarase is present in *Pythium* and *Phytopythium*, and a proteobacterial alcohol dehydrogenase gene is present in multiple *Phytophthora* species (see Table S1 in the supplemental material). The remaining two transferred genes may have more regulatory cytosolic roles in their respective oomycetes species (Table 3), such as regulation of redox activity and neutralization of toxic xenobiotics. Our analysis shows that the transfer of genetic material from bacteria into oomycete lineages is rare but has occurred and that it is another example of cases of HGT between prokaryotes and eukaryotes.

## MATERIALS AND METHODS

### Data set assembly.

The predicted proteomes for seven *Phytophthora* species (*P. capsici*, *P. infestans*, *P. kernoviae*, *P. lateralis*, *P. parasitica*, *P. ramorum*, and *P. sojae*), *Phytopythium vexans*, and six *Pythium* species (*P. aphanidermatum*, *P. arrhenomanes*, *P. irregulare*, *P. iwayami*, *Pythium ultimum* var. *sporangiiferum*, and *P. ultimum* var. *ultimum*) were analyzed for possible bacterium-oomycete HGT events. To ensure a broad taxon sampling for the oomycetes as a whole, we downloaded all available oomycete genome data from public databases. The predicted proteomes of the *Peronosporales* species *Hyaloperonospora arabidopsidis* (71) and *Albugo laibachii* (72); the predicted proteomes of the *Saprolegniales* species *Saprolegnia parasitica* (26), *Saprolegnia diclina*, *Aphanomyces invadans*, and *Aphanomyces astaci* (Broad Institute); and the secretomes of the *Saprolegniales* species *Achyla hypogyna* and *Thraustotheca clavata* (27) were included in our local database. To cover taxon sampling of the stramenopiles, the predicted proteomes of the two diatoms *Phaeodactylum tricornutum* and *Thalassiosira pseudonana* (29, 73) and of the alga *Aureococcus anophagefferens* (74) were also included. In addition to our oocymete and stramenopile data, our database contained all available nonredundant prokaryotic protein data. To construct this portion and reduce redundancy, a representative genome from each prokaryotic species in the full NCBI GenBank database (75) was included. In total, just under 5 million protein sequences from 1,486 prokaryotic genomes were retained. More than 3 million sequences from 212 eukaryotic nuclear genomes, sampling a diverse range of animal, plant, and fungal lineages, were included (see Data Set S1 in the supplemental material).

### Identification of putative bacterium-oomycete HGT events.

Our methods for identifying candidate bacterial HGT genes followed those of Richards et al. (23) in their analysis of fungal HGT genes in the oomycetes. Repetitive and transposable elements were identified and removed from each *Phytophthora* and *Phytopythium*/*Pythium* proteome by performing homology searches against Repbase (76) by the use of tBLASTn (77, 34) with an E value cutoff of 10^−20^ (Table 4). The remaining protein sequences in each oomycete proteome were then further filtered and clustered into groups of paralogs using OrthoMCL (33), with an E value cutoff of 10^−20^ and an inflation value of 1.5 (Table 4). Representative sequences from each group of paralogs, along with unclustered singleton sequences, were retrieved from their respective proteomes. These sequences were then queried against our local database using BLASTp with an E value cutoff of 10^−20^.

**TABLE 4:** Identification of sequences with high bacterial homology corresponding to candidate events of HGT within oomycete genomes

Proteome	Initial size (no. of genes)	Size after Repbase filtering (no. of genes)	No. of OrthoMCL clusters (no. of sequences)	No. of OrthoMCL unclustered sequences	No. of intergenic bacterial hits
*Phytophthora capsici*	19,805	16,169	1,732 (8,879)	7,290	6
*Phytophthora infestans*	18,140	17,013	2,032 (9,459)	7,553	2
*Phytophthora kernoviae*	10,650	10,435	750 (3,244)	7,016	0
*Phytophthora lateralis*	11,635	10,539	880 (4,110)	6,337	14
*Phytophthora parasitica*	20,822	18,640	2,084 (10,153)	8,437	2
*Phytophthora ramorum*	15,743	13,403	1,639 (7,839)	5,564	5
*Phytophthora sojae*	26,584	22,210	2,418 (13,544)	8,666	2
*Phytopythium vexans*	11,958	11,634	1,097 (4,932)	6,702	7
*Pythium aphanidermatum*	12,312	12,002	1,144 (5,129)	6,873	11
*Pythium arrhenomanes*	13,805	13,224	1,221 (5,647)	7,577	18
*Pythium irregulare*	13,805	13,297	1,214 (5,888)	7,409	6
*Pythium iwayami*	14,875	14,279	1,303 (6,185)	8,094	6
*Pythium ultimum* var. *sporangiiferum*	14,096	13,915	917 (4,208)	9,707	13
*Pythium ultimum* var. *ultimum*	15,323	14,780	1,305 (6,661)	8,119	14

Using bespoke python scripting, we identified 106 genes whose homology supported a bacterial transfer into an individual oomycete lineage (encoding proteins whose first hit outside their own genus was bacterial) and retrieved them for a second round of OrthoMCL clustering to remove redundancy in our datasets for each genus (Table 4). All retrieved protein sequences were clustered into groups of orthologs using OrthoMCL with an E value cutoff of 10^−20^ and an inflation value of 1.5 (Table 2). A total of 64 representative and singleton sequences from these datasets were then queried against our local database using BLASTp with an E value cutoff of 10^−20^ and an arbitrary limit for maximum hits per query sequence. The corresponding gene family for each candidate HGT gene was constructed from our BLASTp results.

### Phylogenetic reconstruction of putative bacterium-oomycete HGT events.

A total of 64 candidate HGT gene families were aligned using MUSCLE (78), and best-fit amino acid replacement models were selected for each alignment using ProtTest (38). Maximum-likelihood phylogenetic reconstruction for each alignment was carried out using PhyML (79) with 100 bootstrap replicates. Each phylogenetic tree was visualized and annotated with GenBank data using bespoke python scripting and iTOL (80). Additional phylogenetic analysis using consensus network methods was carried out using SplitsTree (57).

### Analysis of bacterial contamination and taxon sampling in putative bacterium-oomycete HGT families.

Seed genes and their directly adjacent gene were examined for their particular homology to determine whether candidate HGT genes were not simply the result of bacterial contamination of genomes along particular contigs or scaffolds. For each seed gene arising from *P. capsici*, the genomic location of that gene was identified by querying its corresponding protein sequence against the JGI *P. capsici* database (http://genome.jgi.doe.gov/PhycaF7) using tBLASTn with an E value cutoff of 10^−4^. Homology data for each seed gene and their adjacent genes were provided by the JGI *P. capsici* genome browser (see Table S4 in the supplemental material). For each *Pythium* seed gene, the genomic location of the gene was identified by querying the corresponding protein sequence against the genomic scaffolds of the source species using tBLASTn with an E value cutoff of 10^−4^, and then the seed gene’s corresponding protein sequence and its two adjacent protein sequences were queried against the NCBI’s nonredundant protein sequence database using BLASTp with an E value cutoff of 10^−20^ (see Table S4).

For studies of HGT in eukaryotes, particularly transfer between prokaryotes and eukaryotes, it is essential that genomic data cover as broad a range of taxa as possible to prevent as much as possible the introduction of bias into analysis and thus reduce the likelihood of obtaining false transfer events (36, 37). Comparison of the taxon sampling in our database with the NCBI data was performed by searching each seed gene’s protein sequence against the NCBI nonredundant protein sequence database using BLASTp with an E value cutoff of 10^−20^. The seed sequence and its homologs were aligned in MUSCLE, neighbor-joining trees were constructed in QuickTree (81) using 100 bootstrap replicates, and each tree was annotated with GenBank data using bespoke python scripting (not shown). Maximum-likelihood HGT phylogenies whose topology conflicted substantially with their corresponding neighbor-joining tree due to differences in taxon sampling were excluded from further analysis.

### Characterization and functional annotation of putative bacterium-oomycete HGT families.

For the remaining putative HGT families, bespoke python scripting was used to calculate the sequence length, GC content, and exon number of each oomycete gene present. The average sequence length, GC content, and exon number for each *Phytophthora*, *Phytopythium*, and *Pythium* genome were also calculated (data not shown). Multivariate codon usage analysis of each genome was carried out using GCUA (82) (see Fig. S2 in the supplemental material). To compare the properties of each putative HGT family with those of homologs in their potential bacterial donor, multivariate codon usage analysis of the genome of a representative potential donor as well as the relevant seed oomycete gene was also carried out using GCUA. Additionally, the sequence length and GC content of one or more bacterial sister genes were calculated using bespoke python scripting (see Table S2). Optimal local alignments of each seed protein sequence against a representative bacterial sister gene were generated using CLUSTAL Omega (83) (see Table S3). The putative function of each putative HGT family was annotated by performing initial Pfam homology searches of each seed protein sequence (84) (see Data Set S1) with an E value cutoff of 10^−4^ and BLAST homology searches against the NCBI’s nonredundant protein database with an E value cutoff of 10^−20^. To complement these initial annotations, each seed protein sequence was then analyzed in InterProScan (85). Signal peptide analysis and subcellular localization prediction analysis for each seed protein sequence were carried out using SignalP and TargetP, respectively (53, 54), with the default parameters.
