# Supplementary material for: Systematic Search for Evidence of Interdomain Horizontal Gene Transfer from Prokaryotes to Oomycete Lineages
Source: mSphere. 2016 Sep 14;1(5):e00195-16. doi: 10.1128/mSphere.00195-16 (PMC5023847; doi:10.1128/mSphere.00195-16)
Supplement: Table S1 [file sph005162148st6.docx]

**Table S1.** Genetic characteristics of putative bacterial HGT genes in oomycete genomes and comparison with mean genetic characteristics of corresponding genome. Refer to corresponding figure for phylogenetic tree. *Seed gene in italics.*

| **Sequence** | **GC (%)** | **Mean GC** | **Length (bp)** | **Mean length** | **Exons** | **Mean exons** |
| --- | --- | --- | --- | --- | --- | --- |
| ***Pythium* spp. */ Phytopythium* class II fumarase (Figure 1)** | | | | | | |
| PYAP\|001813 | 57.28 | 55.96 | 1395.00 | 1471.15 | 1 | 2.33 |
| PYAR\|002667 | 60.99 | 60.22 | 2730.00 | 1342.37 | 4 | 2.65 |
| PYIR\|004913 | 60.84 | 58.67 | 1407.00 | 1497.05 | 2 | 2.88 |
| PYIW\|009108 | 52.38 | 55.99 | 2268.00 | 1143.85 | 7 | 2.76 |
| *PYUS\|000774* | 61.95 | 55.99 | 1017.00 | 1143.85 | 5 | 3.67 |
| PYUU\|003434 | 62.63 | 56.72 | 1389.00 | 1314.79 | 2 | 2.62 |
| PYVX\|000932 | 63.20 | 62.25 | 1386.00 | 1576.47 | 2 | 2.93 |
| ***Pythium* spp. NmrA-like quinone oxidoreductase (Figure 2)** | | | | | | |
| *PYAP\|009189* | 56.70 | 55.96 | 903.00 | 1471.15 | 1 | 2.33 |
| PYAP\|010623 | 56.20 | 55.96 | 822.00 | 1471.15 | 2 | 2.33 |
| PYAP\|012028 | 61.98 | 55.96 | 1131.00 | 1471.15 | 1 | 2.33 |
| PYAR\|009100 | 60.74 | 60.22 | 759.00 | 1342.37 | 3 | 2.65 |
| PYAR\|009101 | 65.34 | 60.22 | 906.00 | 1342.37 | 1 | 2.65 |
| PYAR\|010513 | 64.04 | 60.22 | 801.00 | 1342.37 | 2 | 2.65 |
| PYIR\|011515 | 61.11 | 58.67 | 900.00 | 1497.05 | 2 | 2.88 |
| PYIW\|000026 | 59.83 | 55.99 | 1170.00 | 1143.85 | 1 | 2.76 |
| PYIW\|006437 | 61.28 | 55.99 | 576.00 | 1143.85 | 1 | 2.76 |
| PYIW\|007663 | 57.10 | 55.99 | 753.00 | 1143.85 | 1 | 2.76 |
| PYIW\|011798 | 49.08 | 55.99 | 1143.00 | 1143.85 | 2 | 2.76 |
| PYUU\|001009 | 61.15 | 56.72 | 924.00 | 1314.79 | 1 | 2.62 |
| ***Phytophthora capsici* epoxide hydrolase (Figure 4)** | | | | | | |
| *PHYC\|001503* | 53.15 | 52.93 | 873.00 | 1028.23 | 1 | 2.20 |
| PHYC\|019423 | 52.53 | 52.93 | 1106.00 | 1028.23 | 8 | 2.20 |

| **Sequence** | **GC (%)** | **Mean GC** | **Length (bp)** | **Mean length** | **Exons** | **Mean exons** |
| --- | --- | --- | --- | --- | --- | --- |
| ***Pythium* spp. SnoaL-like protein (Figure 3)** | | | | | | |
| *PYAP\|012127* | 63.84 | 55.96 | 921.00 | 1471.15 | 1 | 2.33 |
| PYIR\|008111 | 60.43 | 58.67 | 417.00 | 1497.05 | 2 | 2.88 |
| PYIR\|012605 | 62.66 | 58.67 | 774.00 | 1497.05 | 1 | 2.88 |
| PYIR\|013034 | 60.36 | 58.67 | 825.00 | 1497.05 | 1 | 2.88 |
| PYIR\|013075 | 60.75 | 58.67 | 777.00 | 1497.05 | 1 | 2.88 |
| PYIR\|013356 | 61.97 | 58.67 | 831.00 | 1497.05 | 1 | 2.88 |
| PYIW\|006880 | 54.92 | 55.99 | 1098.00 | 1143.85 | 2 | 2.76 |
| PYIW\|007179 | 57.75 | 55.99 | 258.00 | 1143.85 | 1 | 2.76 |
| PYIW\|007664 | 59.04 | 55.99 | 354.00 | 1143.85 | 1 | 2.76 |
| PYIW\|011895 | 55.87 | 55.99 | 2214.00 | 1143.85 | 3 | 2.76 |
| PYUS\|013198 | 53.81 | 55.99 | 381.00 | 1143.85 | 1 | 3.67 |
| PYUS\|013248 | 52.76 | 55.99 | 453.00 | 1143.85 | 1 | 3.67 |
| PYUU\|001934 | 54.71 | 56.72 | 912.00 | 1314.79 | 1 | 2.62 |
| PYUU\|006243 | 58.69 | 56.72 | 915.00 | 1314.79 | 1 | 2.62 |
| PYUU\|012802 | 61.29 | 56.72 | 912.00 | 1314.79 | 1 | 2.62 |
| PYUU\|012810 | 53.29 | 56.72 | 897.00 | 1314.79 | 1 | 2.62 |
| PYUU\|012826 | 61.29 | 56.72 | 465.00 | 1314.79 | 1 | 2.62 |
| ***Phytophthora* spp. alcohol dehydrogenase (Figure 5)** | | | | | | |
| *PHYC\|018145* | 55.34 | 52.93 | 918.00 | 1028.23 | 2 | 2.20 |
| PHYI\|007077 | 56.22 | 53.81 | 1005.00 | 1310.65 | 1 | 2.85 |
| PHYK\|007822 | 57.41 | 54.13 | 1005.00 | 1065.19 | 1 | 3.60 |
| PHYR\|002043 | 58.11 | 58.53 | 1005.00 | 1428.62 | 1 | 2.58 |
| PHYR\|002241 | 57.81 | 58.53 | 1005.00 | 1428.62 | 1 | 2.58 |
| PHYR\|010535 | 57.41 | 58.53 | 1005.00 | 1428.62 | 1 | 2.58 |
| PHYS\|011254 | 58.98 | 58.02 | 1080.00 | 1181.45 | 1 | 2.39 |
