# Supplementary material for: Systematic Search for Evidence of Interdomain Horizontal Gene Transfer from Prokaryotes to Oomycete Lineages
Source: mSphere. 2016 Sep 14;1(5):e00195-16. doi: 10.1128/mSphere.00195-16 (PMC5023847; doi:10.1128/mSphere.00195-16)
Supplement: Table S2 [file sph005162148st7.docx]

**Table S2.** Characterisatics of potenital sister genes to candidate HGT genes from potential donor bacterial species. Refer to corresponding figure for phylogenetic tree.

| **Tree** | **Bacterial homolog** | **Species** | **Length (bp)** | **GC (%)** | **Species GC (%)** |
| --- | --- | --- | --- | --- | --- |
| **Figure 1** | YP_005440376 | *Caldilinea aerophila* DSM 14535 = NBRC 104270 | 1388 | 60.37 | 58.80 |
| **Figure 2** | YP_003377641 | *Xanthomonas albilineans* GPE PC73 | 908 | 67.29 | 62.90 |
| **Figure 2** | YP_005436915 | *Rubrivivax gelatinosus* IL144 | 890 | 75.51 | 71.20 |
| **Figure 2** | YP_001208040 | *Bradyrhizobium* sp. ORS 278 | 869 | 68.58 | 65.50 |
| **Figure 2** | YP_001237736 | *Bradyrhizobium* sp. BTAi1 | 869 | 68.35 | 64.79 |
| **Figure 2** | YP_521565 | *Rhodoferax ferrireducens* T118 | 890 | 65.51 | 59.62 |
| **Figure 3** | YP_007933011 | *Streptomyces fulvissimus* DSM 40593 | 395 | 71.40 | 71.50 |
| **Figure 3** | YP_006241405 | *Streptomyces hygroscopicus* subsp. *jinggangensis* 5008 | 461 | 59.00 | 71.85 |
| **Figure 3** | YP_006241332 | *Streptomyces hygroscopicus* subsp. *jinggangensis* 5008 | 371 | 59.59 | 71.85 |
| **Figure 3** | YP_007526282 | *Streptomyces davawensis* JCM 4913 | 737 | 67.03 | 70.59 |
| **Figure 4** | YP_001766910 | *Methylobacterium radiotolerans* JCM 2831 | 965 | 67.15 | 71.04 |
| **Figure 5** | YP_007616234 | *Sphingomonas* sp. MM-1 | 1010 | 69.80 | 66.69 |
| **Figure 5** | YP_001263784 | *Sphingomonas wittichii* RW1 | 1013 | 73.15 | 67.90 |
