# Supplementary material for: Systematic Search for Evidence of Interdomain Horizontal Gene Transfer from Prokaryotes to Oomycete Lineages
Source: mSphere. 2016 Sep 14;1(5):e00195-16. doi: 10.1128/mSphere.00195-16 (PMC5023847; doi:10.1128/mSphere.00195-16)
Supplement: Table S3 [file sph005162148st8.docx]

**Table S3.** Local protein-protein alignments of candidate HGT genes with homologs in potential donor species.

| **Tree** | **Seed** | **Bacterial homolog** | **Identity (%)** | **Similarity (%)** |
| --- | --- | --- | --- | --- |
| **Figure 1** | PYUS\|000774 | YP_005440376 (*C. aerophila*) | 46.4 | 56.5 |
| **Figure 2** | PYAP\|009189 | YP_521565 (*R. ferrireducens*) | 36.3 | 54.0 |
| **Figure 3** | PYAP\|012127 | YP_007526282 (*S. davawensis*) | 37.5 | 58.6 |
| **Figure 4** | PHYC\|001503 | YP_001766910 (*M. radiotolerans*) | 54.5 | 68.2 |
| **Figure 5** | PHYC\|018145 | YP_007616234 (*Sphingomonas* sp. MM-1) | 41.8 | 59.1 |
