# Supplementary material for: Systematic Search for Evidence of Interdomain Horizontal Gene Transfer from Prokaryotes to Oomycete Lineages
Source: mSphere. 2016 Sep 14;1(5):e00195-16. doi: 10.1128/mSphere.00195-16 (PMC5023847; doi:10.1128/mSphere.00195-16)
Supplement: Table S4 [file sph005162148st9.docx]

**Table S4.** Homology analysis of putative HGT sequences and adjacent sequences in oomycete genomes. Purple: *Phytophthora*, green: Bacteria, blue: *Aphanomyces invadans*, yellow: *Strongylocentrotus purpuratus*.

| **Left flanking sequence** | | **Seed sequence** | | **Right flanking sequence** | |
| --- | --- | --- | --- | --- | --- |
| ***Pythium* spp. */ Phytopythium* class II fumarase (Figure 1)** *(Seed: PYUS\|000774)* | | | | | |
| **Contig** | 27 | **Contig** | 27 | **Contig** | 27 |
| **Coordinates** | 57345-58979 | **Coordinates** | 59555-61047 | **Coordinates** | 61383-62666 |
| **NCBI Best Hit** | *P. parasitica* | **NCBI Best Hit** | *C. aerophila* | **NCBI Best Hit** | *P. parasitica* |
| ***Pythium* spp. NmrA-like quinone oxidoreductase (Figure 2)** *(Seed: PYAP\|009189)* | | | | | |
| **Scaffold** | 558 | **Scaffold** | 559 | **Scaffold** | 559 |
| **Coordinates** | 19267-20010 | **Coordinates** | 731-1630 | **Coordinates** | 2606-2860 |
| **NCBI Best Hit** | *P. infestans* | **NCBI Best Hit** | *R. gelatinosus* | **NCBI Best Hit** | *P. infestans* |
| ***Pythium* spp. SnoaL-like protein (Figure 3)** *(Seed: PYAP\|012127)* | | | | | |
| **Scaffold** | 1511 | **Scaffold** | 1511 | **Scaffold** | 1513 |
| **Coordinates** | 50-760 | **Coordinates** | 2127-3047 | **Coordinates** | 1000-2916 |
| **NCBI Best Hit** | *A. invadans* | **NCBI Best Hit** | *S. yerevanensis* | **NCBI Best Hit** | *P. parasitica* |
| ***Phytophthora capsici* epoxide hydrolase (Figure 4)** *(Seed: PHYC\|001503)* | | | | | |
| **Scaffold** | 10 | **Scaffold** | 10 | **Scaffold** | 10 |
| **Coordinates** | 1086791-1088105 | **Coordinates** | 1089394-1090266 | **Coordinates** | 1090611-1092539 |
| **JGI Best Hit** | *P. sojae* | **JGI Best Hit** | *A. vinelandii* | **JGI Best Hit** | *P. ramorum* |
| ***Phytophthora* spp. alcohol dehydrogenase (Figure 5)** *(Seed: PHYC\|018145)* | | | | | |
| **Scaffold** | 25 | **Scaffold** | 25 | **Scaffold** | 25 |
| **Coordinates** | 616564-617025 | **Coordinates** | 617829-618823 | **Coordinates** | 619122-620910 |
| **JGI Top Hit** | *S. purpuratus* | **JGI Top Hit** | *P. sojae* | **JGI Top Hit** | *P. ramorum* |
