# Supplementary material for: Systematic Search for Evidence of Interdomain Horizontal Gene Transfer from Prokaryotes to Oomycete Lineages
Source: mSphere. 2016 Sep 14;1(5):e00195-16. doi: 10.1128/mSphere.00195-16 (PMC5023847; doi:10.1128/mSphere.00195-16)
Supplement: Figure S1 [file sph005162148sf1.pdf]

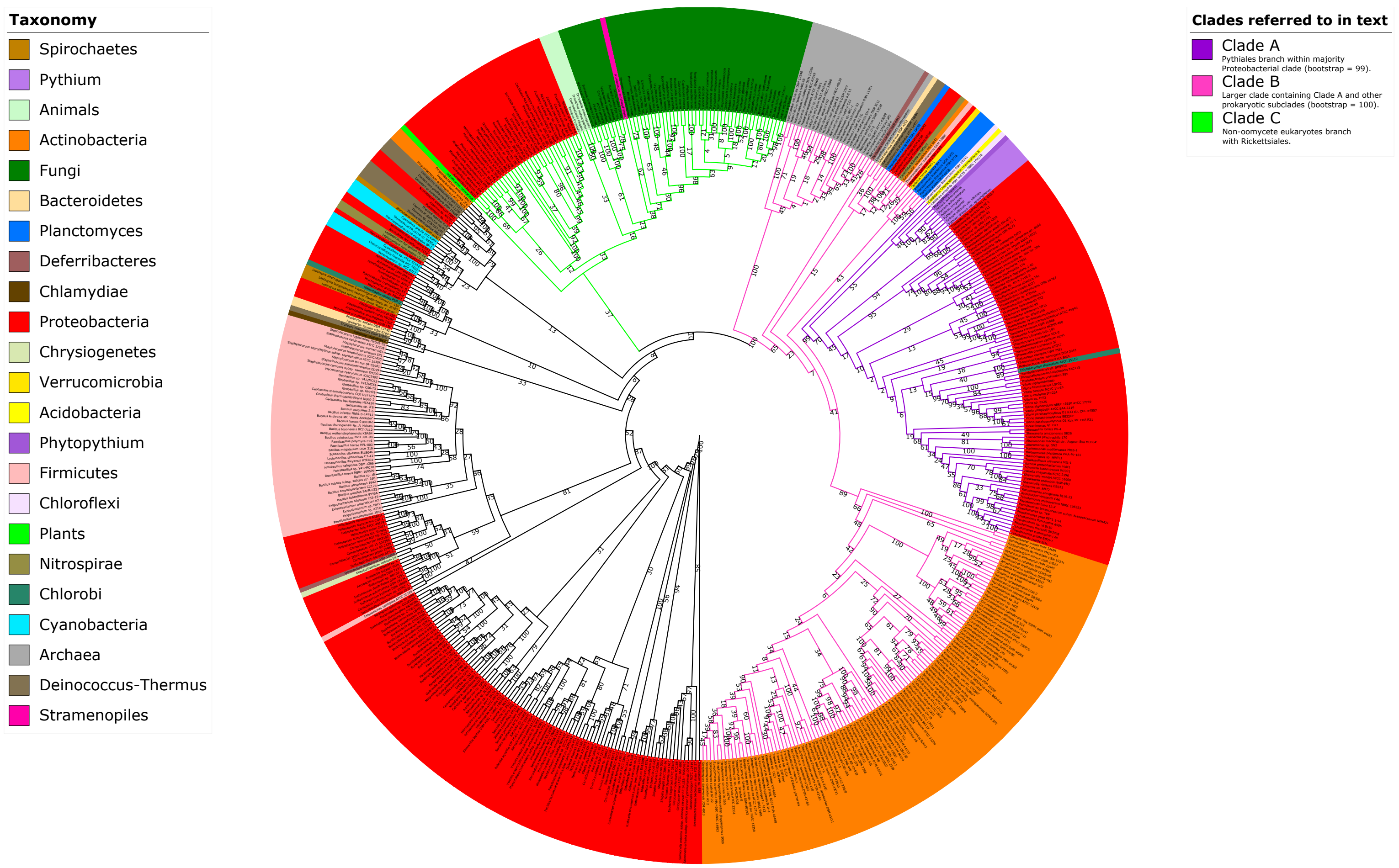
Taxonomy
Spirochaetes
Pythium
Animals
Actinobacteria
Fungi
Bacteroidetes
Planctomyces
Deferribacteres
Chlamydiae
Proteobacteria
Chrysiogenetes
Verrucomicrobia
Acidobacteria
Phytopythium
Firmicutes
Chloroflexi
Plants
Nitrospirae
Chlorobi
Cyanobacteria
Archaea
Deinococcus-Thermus
Stramenopiles
Clades referred to in text
Clade A
Pythiales branch within majority Proteobacterial clade (bootstrap = 99).
Clade B
Larger clade containing Clade A and other prokaryotic subclades (bootstrap = 100).
Clade C
Non-oomycete eukaryotes branch with Rickettsiales.
