# Supplementary material for: Systematic Search for Evidence of Interdomain Horizontal Gene Transfer from Prokaryotes to Oomycete Lineages
Source: mSphere. 2016 Sep 14;1(5):e00195-16. doi: 10.1128/mSphere.00195-16 (PMC5023847; doi:10.1128/mSphere.00195-16)
Supplement: Figure S2 [file sph005162148sf2.pdf]

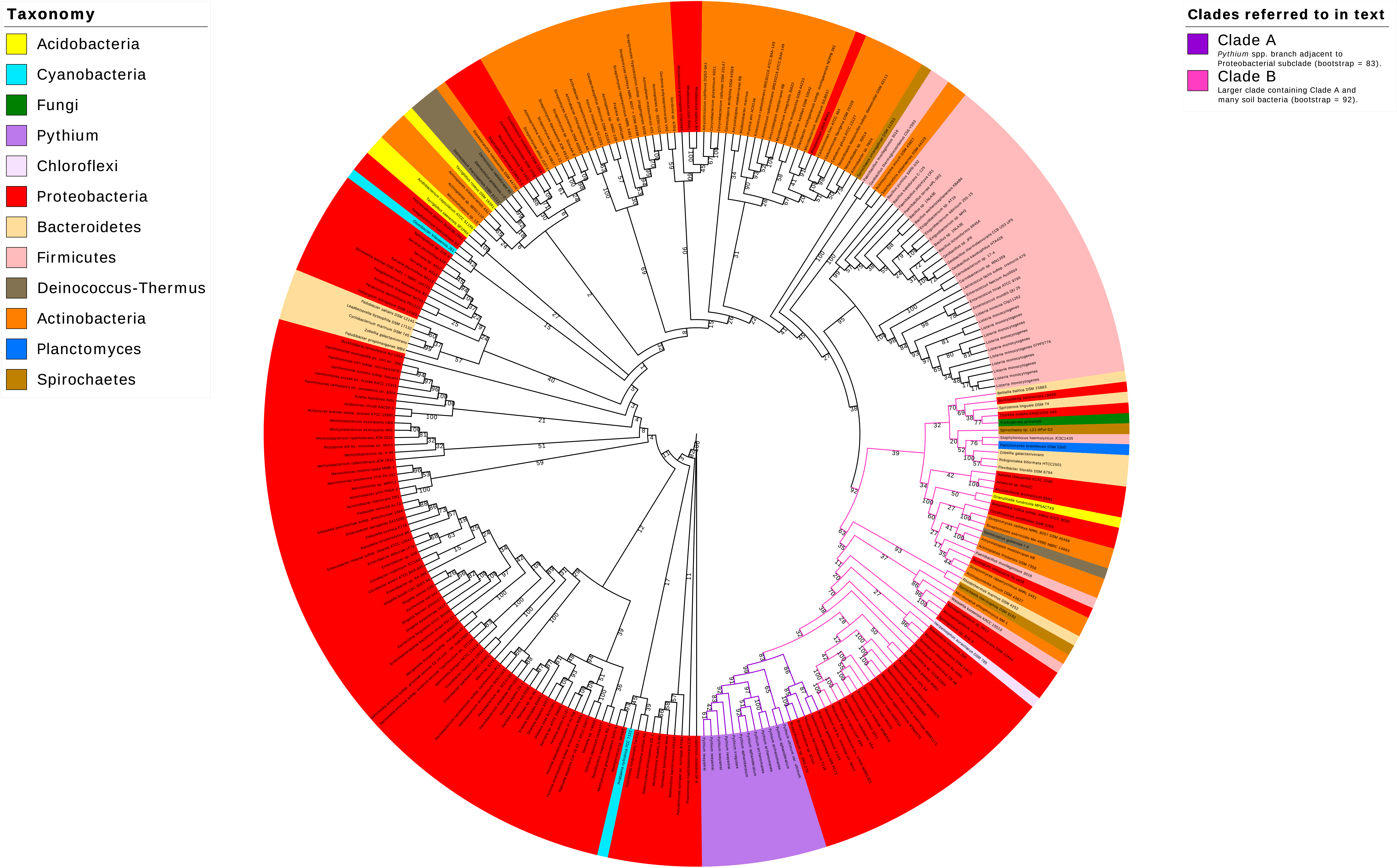
Taxonomy
Acidobacteria
Cyanobacteria
Fungi
Pythium
Chloroflexi
Proteobacteria
Bacteroidetes
Firmicutes
Deinococcus-Thermus
Actinobacteria
Planctomyces
Spirochaetes
Clades referred to in text
Clade A
Pythium spp. branch adjacent to Proteobacterial subclade (bootstrap = 83).
Clade B
Larger clade containing Clade A and many soil bacteria (bootstrap = 92).
