# Supplementary material for: Systematic Search for Evidence of Interdomain Horizontal Gene Transfer from Prokaryotes to Oomycete Lineages
Source: mSphere. 2016 Sep 14;1(5):e00195-16. doi: 10.1128/mSphere.00195-16 (PMC5023847; doi:10.1128/mSphere.00195-16)
Supplement: Figure S3 [file sph005162148sf3.pdf]

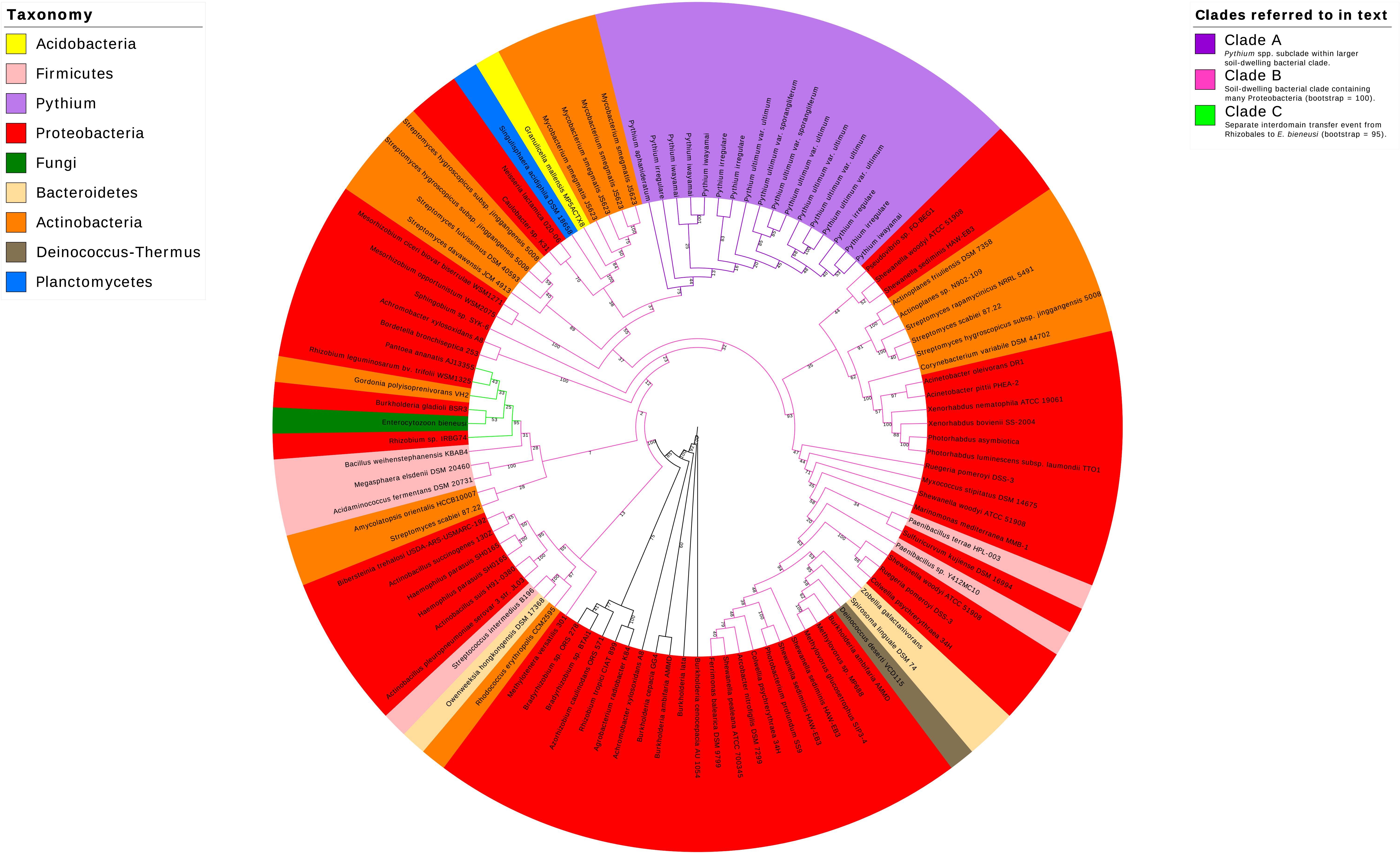

Taxonomy
Acidobacteria
Firmicutes
Pythium
Proteobacteria
Fungi
Bacteroidetes
Actinobacteria
Deinococcus-Thermus
Planctomycetes
Clades referred to in text
Clade A
Pythium spp. subclade within larger soil-dwelling bacterial clade.
Clade B
Soil-dwelling bacterial clade containing many Proteobacteria (bootstrap = 100).
Clade C
Separate interdomain transfer event from Rhizobales to E. bieneusi (bootstrap = 95).
Pythium aphanidermatum
Pythium irregulare
Pythium iwayamai
Pythium iwayamai
Pythium iwayamai
Pythium irregulare
Pythium irregulare
Pythium ultimum var. ultimum
Pythium ultimum var. sporangiiferum
Pythium ultimum var. sporangiiferum
Pythium ultimum var. ultimum
Pythium ultimum var. ultimum
Pythium ultimum var. ultimum
Pythium irregulare
Pythium irregulare
Pythium iwayamai
Pseudovibrio sp. FO-BEG1
Shewanella woodyi ATCC 51908
Shewanella sediminis HAW-EB3
Actinoplanes friuliensis DSM 7358
Actinoplanes sp. N902-109
Streptomyces rapamycinicus NRRL 5491
Streptomyces scabiei 87.22
Streptomyces hygroscopicus subsp. jinggangensis 5008
Corynebacterium variabile DSM 44702
Acinetobacter oleivorans DR1
Acinetobacter pittii PHEA-2
Xenorhabdus nematophila ATCC 19061
Xenorhabdus bovienii SS-2004
Photorhabdus asymbiotica
Photorhabdus luminescens subsp. laumondii TTO1
Ruegeria pomeroyi DSS-3
Myxococcus stipitatus DSM 14675
Shewanella woodyi ATCC 51908
Marinomonas mediterranea MMB-1
Paenibacillus terrae HPL-003
Sulfuricurvum kujiense DSM 16994
Paenibacillus sp. Y412MC10
Shewanella woodyi ATCC 51908
Ruegeria pomeroyi DSS-3
Colwellia psychrerythraea 34H
Zobellia galactanivorans
Spirosoma linguale DSM 74
Deinococcus deserti VCD115
Burkholderia ambifaria AMMD
Methylovorus sp. MP688
Methylovorus glucosetrophus SIP3-4
Shewanella sediminis HAW-EB3
Shewanella sediminis HAW-EB3
Photobacterium profundum SS9
Colwellia psychrerythraea 34H
Arcobacter nitrofigilis DSM 7299
Shewanella pealeana ATCC 700345
Ferrimonas balearica DSM 9799
Burkholderia cenocepacia AU 1054
Burkholderia lata
Burkholderia ambifaria AMMD
Burkholderia cepacia GG4
Achromobacter xylosoxidans A8
Agrobacterium radiobacter K84
Rhizobium tropici CIAT 899
Azorhizobium caulinodans ORS 571
Bradyrhizobium sp. BTAi1
Bradyrhizobium sp. ORS 278
Methylotenera versatilis 301
Rhodococcus erythropolis CCM2595
Owenweeksia hongkongensis DSM 17368
Streptococcus intermedius B196
Actinobacillus pleuropneumoniae serovar 3 str. JL03
Actinobacillus suis H91-0380
Haemophilus parasuis SH0165
Haemophilus parasuis SH0165
Actinobacillus succinogenes 130Z
Bibersteinia trehalosi USDA-ARS-USMARC-192
Streptomyces scabiei 87.22
Amycolatopsis orientalis HCCB10007
Acidaminococcus fermentans DSM 20731
Megasphaera elsdenii DSM 20460
Bacillus weihenstephanensis KBAB4
Rhizobium sp. IRBG74
Enterocytozoon bieneusi
Burkholderia gladioli BSR3
Gordonia polyisoprenivorans VH2
Rhizobium leguminosarum bv. trifolii WSM1325
Pantoea ananatis AJ13355
Bordetella bronchiseptica 253
Achromobacter xylosoxidans A8
Sphingobium sp. SYK-6
Mesorhizobium opportunistum WSM2075
Mesorhizobium ciceri biovar biserrulae WSM1271
Streptomyces davawensis JCM 4913
Streptomyces fulvissimus DSM 40593
Streptomyces hygroscopicus subsp. jinggangensis 5008
Streptomyces hygroscopicus subsp. jinggangensis 5008
Neisseria lactamica 020-06
Caulobacter sp. K31
Singulisphaera acidiphila DSM 18658
Granulicella mallensis MP5ACTX8
Mycobacterium smegmatis JS623
Mycobacterium smegmatis JS623
Mycobacterium smegmatis JS623
Mycobacterium smegmatis JS623
