# Supplementary material for: Systematic Search for Evidence of Interdomain Horizontal Gene Transfer from Prokaryotes to Oomycete Lineages
Source: mSphere. 2016 Sep 14;1(5):e00195-16. doi: 10.1128/mSphere.00195-16 (PMC5023847; doi:10.1128/mSphere.00195-16)
Supplement: Figure S4 [file sph005162148sf4.pdf]

(A)

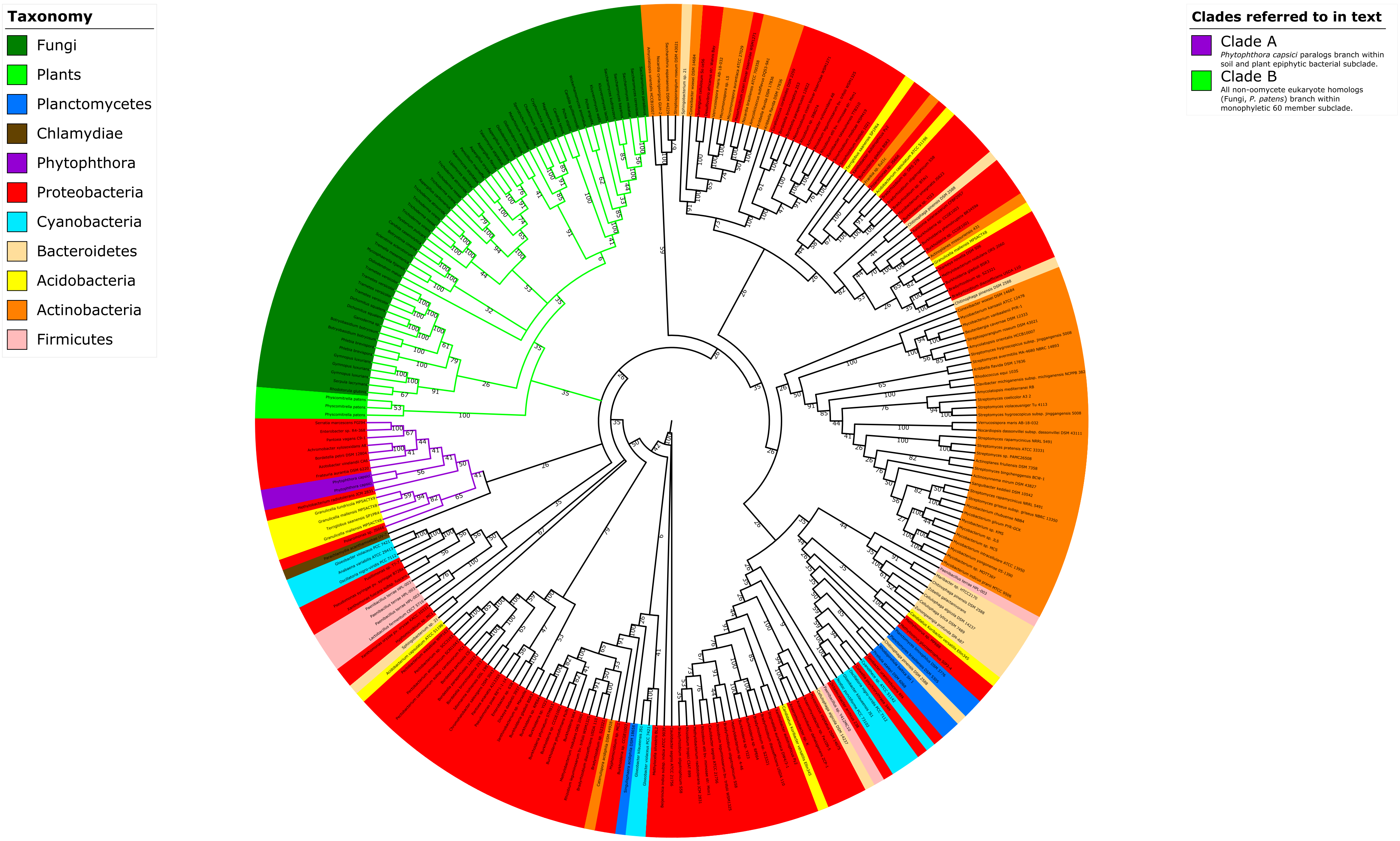
Taxonomy
Fungi
Plants
Planctomycetes
Chlamydiae
Phytophthora
Proteobacteria
Cyanobacteria
Bacteroidetes
Acidobacteria
Actinobacteria
Firmicutes
Clades referred to in text
Clade A
Phytophthora capsici paralogs branch within soil and plant epiphytic bacterial subclade.
Clade B
All non-oomycete eukaryote homologs (Fungi, P. patens) branch within monophyletic 60 member subclade.

(B)

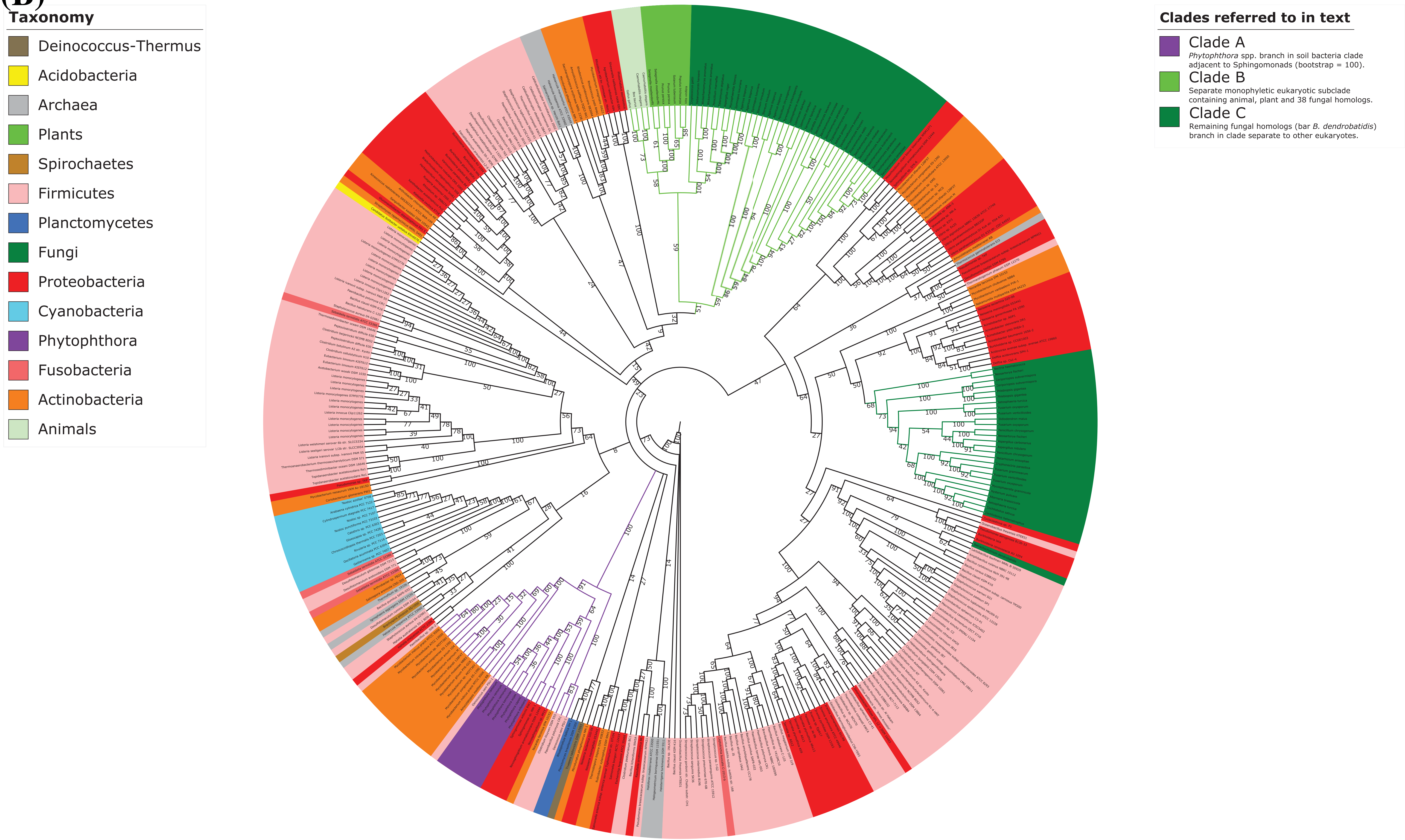
Taxonomy
Deinococcus-Thermus
Acidobacteria
Archaea
Plants
Spirochaetes
Firmicutes
Planctomycetes
Fungi
Proteobacteria
Cyanobacteria
Phytophthora
Fusobacteria
Actinobacteria
Animals
Clades referred to in text
Clade A
Phytophthora spp. branch in soil bacteria clade adjacent to Sphingomonads (bootstrap = 100).
Clade B
Separate monophyletic eukaryotic subclade containing animal, plant and 38 fungal homologs.
Clade C
Remaining fungal homologs (bar B. dendrobatidis) branch in clade separate to other eukaryotes.
