# Supplementary figures and images for: Systematic Search for Evidence of Interdomain Horizontal Gene Transfer from Prokaryotes to Oomycete Lineages
Source: mSphere. 2016 Sep 14;1(5):e00195-16. doi: 10.1128/mSphere.00195-16 (PMC5023847; doi:10.1128/mSphere.00195-16)

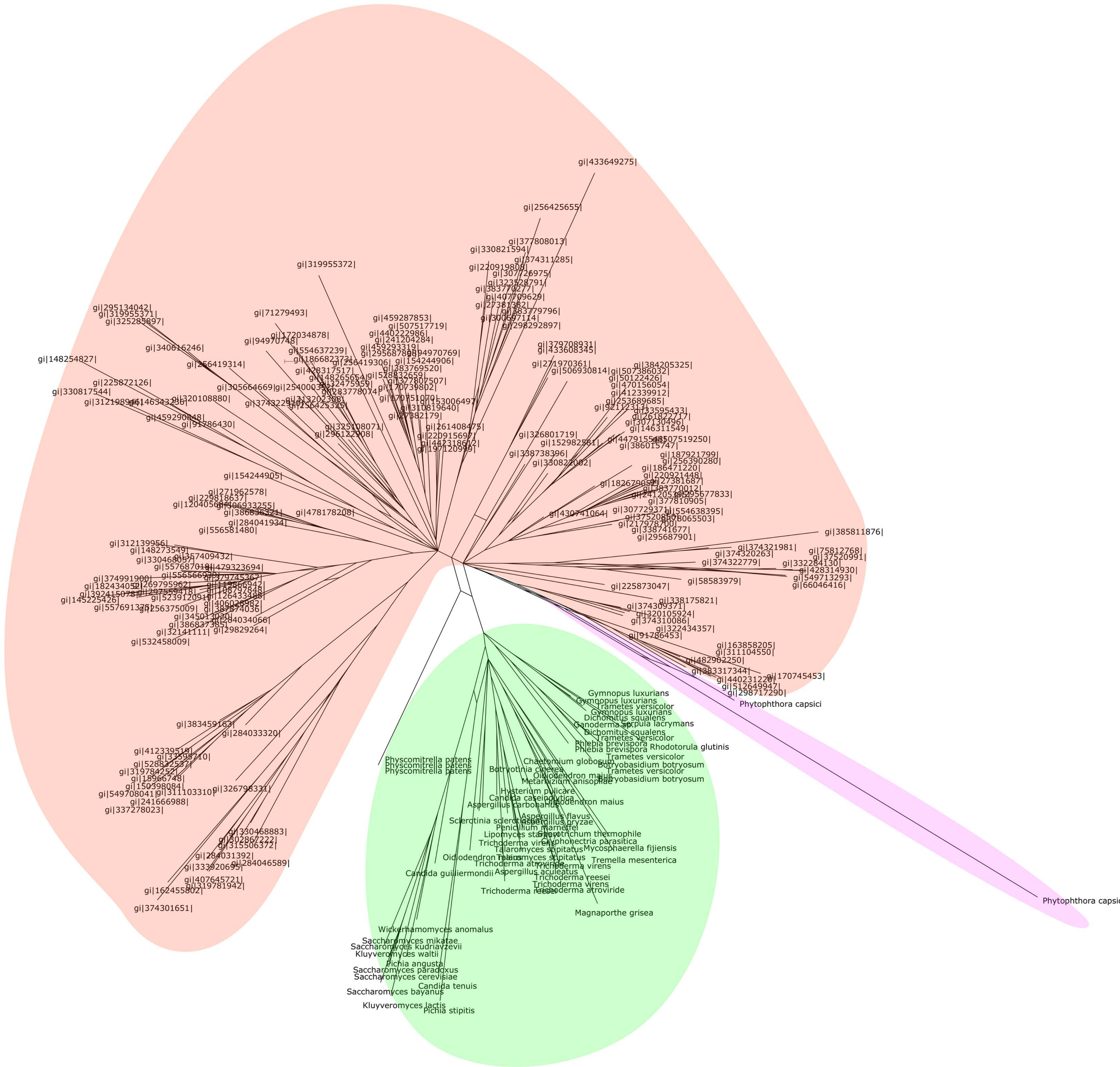

Supplement: Figure S5 [file sph005162148sf5.pdf]
